# Flexible characterization of animal movement pattern using net squared displacement and a latent state model

**DOI:** 10.1186/s40462-016-0080-y

**Published:** 2016-06-01

**Authors:** Guillaume Bastille-Rousseau, Jonathan R. Potts, Charles B. Yackulic, Jacqueline L. Frair, E. Hance Ellington, Stephen Blake

**Affiliations:** Department of Environmental and Forest Biology, State University of New York, College of Environmental Science and Forestry, Syracuse, 13210 NY USA; Roosevelt Wild Life Station, State University of New York, College of Environmental Science and Forestry, Syracuse, 13210 NY USA; School of Mathematics and Statistics, University of Sheffield, Hicks Building, Hounsfield Road, Sheffield, S3 7RH UK; U.S. Geological Survey, Southwest Biological Science Center, Grand Canyon Monitoring and Research Center, Flagstaff, 86001 AZ USA; School of Environment and Natural Resources, Ohio State University, Columbus, 43210 OH USA; Saint Louis Zoo, WildCare Institute, 1 Government Drive, St. Louis, 63110 MO USA; Max Planck Institute for Ornithology, Radolfzell, Germany; Whitney Harris World Ecology Center, University of Missouri-St. Louis, St. Louis, 63121 MO USA; Department of Biology, Washington University in St. Louis, St. Louis, 63130 MO USA; Charles Darwin Foundation, Puerto Ayora, Galapagos Ecuador

**Keywords:** Bayesian clustering, Mixture model, Giant Galapagos tortoises (*Chelonoidis* sp.), Migration, Resident, Discrete latent state

## Abstract

**Background:**

Characterizing the movement patterns of animals is an important step in understanding their ecology. Various methods have been developed for classifying animal movement at both coarse (e.g., migratory vs. sedentary behavior) and fine (e.g., resting vs. foraging) scales. A popular approach for classifying movements at coarse resolutions involves fitting time series of net-squared displacement (NSD) to models representing different conceptualizations of coarse movement strategies (i.e., migration, nomadism, sedentarism, etc.). However, the performance of this method in classifying actual (as opposed to simulated) animal movements has been mixed. Here, we develop a more flexible method that uses the same NSD input, but relies on an underlying discrete latent state model. Using simulated data, we first assess how well patterns in the number of transitions between modes of movement and the duration of time spent in a mode classify movement strategies. We then apply our approach to elucidate variability in the movement strategies of eight giant tortoises (*Chelonoidis* sp.) using a multi-year (2009–2014) GPS dataset from three different Galapagos Islands.

**Results:**

With respect to patterns of time spent and the number of transitions between modes, our approach out-performed previous efforts to distinguish among migration, dispersal, and sedentary behavior. We documented marked inter-individual variation in giant tortoise movement strategies, with behaviors indicating migration, dispersal, nomadism and sedentarism, as well as hybrid behaviors such as “exploratory residence”.

**Conclusions:**

Distilling complex animal movement into discrete modes remains a fundamental challenge in movement ecology, a problem made more complex by the ever-longer duration, ever-finer resolution, and gap-ridden trajectories recorded by GPS devices. By clustering into modes, we derived information on the time spent within one mode and the number of transitions between modes which enabled finer differentiation of movement strategies over previous methods. Ultimately, the techniques developed here address limitations of previous approaches and provide greater insights with respect to characterization of movement strategies across scales by more fully utilizing long-term GPS telemetry datasets.

**Electronic supplementary material:**

The online version of this article (doi:10.1186/s40462-016-0080-y) contains supplementary material, which is available to authorized users.

## Background

Understanding the drivers and implications of an organism’s movement remains a fundamental challenge in ecology [1]. Movement has important repercussions for an individual’s fitness and can influence the structure and function of populations, communities and ecosystems [1]. Movement can be classified at multiple temporal and spatial scales [2]. For example, movement patterns at coarse temporal scales (e.g., annual) can be classified in terms of broad strategies (e.g., migration, dispersal, residency, or nomadism; hereafter referred to as “movement strategies”), while variation at finer temporal scales is frequently thought of in terms of latent or behavioral modes (e.g., “encamped” vs. “exploratory” [3, 4]; hereafter referred to as “modes”). Movement strategies at even finer temporal scales can be envisioned when animals transition among different behavioral states (e.g., resting, moving, foraging, hereafter referred to as “states”). Recent studies have shown that broad-scale movement strategies vary substantially among and within species, and even among individuals within a population [5–8].

The benefits of different strategies and the rates of transitions between behavioral modes can differ depending on the spatial and temporal structuring of resources [5, 9, 10]. Linking dynamic resource distributions to movement patterns at different spatial and temporal resolutions remains a critical step toward understanding the drivers of movement decisions. Thus, classifying movement strategies is a logical first step in the analysis of animal movement [1]. While much research has focused on quantifying patterns of animal movement at the scales of movement strategies, modes, or states independently, to date few analytical approaches have applied methods oriented toward a specific movement scale to understanding movement at other scales. [11, 12]. For example, approaches for identifying strategies have generally relied on relatively rigid parametric models in comparison to approaches for identifying movement modes or behavioral state (e.g., [7, 13, 14]). As a result, our understanding of the drivers of movement patterns remains limited in part by the lack of flexible approaches to quantify large-scale animal movement strategies.

**Fig. 1:**
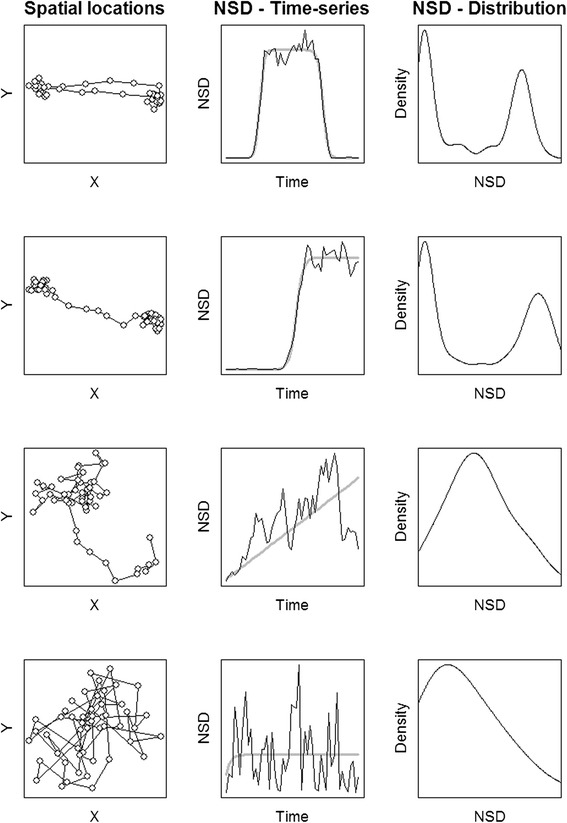
Depiction of four movement strategies: Migration, dispersal, nomadism, and sedentarism. Legend: Spatial locations in the X-Y plane are shown along with the temporal pattern in net squared displacement (*NSD, thin line*) and theoretical expectation (*grey thick line*) and the frequency distribution of NSD values for four given movement strategies: migration (*first row*), dispersal (*second row*), nomadism (*third row*), and sedentarism (*fourth row*)

A popular approach to identifying movement strategies focuses on the net squared displacement (NSD) of individuals over time (but see also [15]) – part of a family of metrics known as “synthetic statistics” that capture key properties of animal movement [16, 17]. NSD measures the square of the Euclidean distance between the starting location of a movement path and each subsequent location. Time-series of NSD values are characteristic of individual movement trajectories. When averaged over time, NSD time-series can be summarized as the mean squared displacement. Mean squared displacement is used to quantify the diffusive spread of particles, or animals, over time and space. Distinct patterns in NSD time-series are theoretically expected from specific movement strategies, and parametric models have been suggested to help elucidate those patterns (e.g., a double-sigmoid curve represents migration; Fig. 1, first row).

Efforts to classify movement strategies are typically organized around the concepts of sedentarism, dispersal, nomadism, and migration [7, 13, 14]. Bunnefeld et al. [14] fit alternative models to NSD time-series from an individual, with models corresponding to idealized movement strategies (double-sigmoid = migration, sigmoid = dispersal, linear = nomadism, asymptotic = sedentarism; grey lines in Fig. 1), and used an information criterion (e.g., AIC; [18]) to assign the most likely movement strategy. Expanding on that approach, Börger & Fryxell [13] fit non-linear mixed models to account for non-independence in the time-series of animal locations, and “borrowed” strength by model fitting pooled animals. While these approaches have performed well in specific instances [7, 13, 14], empirical animal trajectories often defy classification, showing greater variability than the anticipated stereotypical ideal. Some applications of the Bunnefeld et al. [14] approach have produced high classification error in classifying movement strategies or aberrant results based on visual validation [6, 8, 15, 19, 20]. NSD approaches also suffer from inherent statistical problems related to temporal autocorrelation (which we develop in the Discussion below), that limit their applicability. Lastly, they rely on fragmenting the data into independent subsets of homogenous duration (e.g., 1 year of data) that correspond to model expectations, rather than being flexible enough to handle a variety of monitoring durations from multiple years of data and the complexity of trajectories from long-lived species.

Here, we integrate the NSD statistic with latent, discrete-state models (a type of hidden Markov model) to improve the flexibility and accuracy of classification of large-scale movement strategies [2, 3]. Latent state models define states in terms of distributions of one or more measured characteristics of a movement path (e.g., step length and turning angles, or in our case NSD) [3, 4]. In addition to quantifying NSD moments for each state, with the number of states defined by the model structure, latent state models also estimate the probability that an animal is in any of the possible states at each point in time. In the simplest versions, the state for each observation (i.e., GPS location) is dependent only on the associated movement characteristics (i.e., the step length and turning angle at that time). However, additional information is available from the temporal dependency of states by modelling transitions between states assuming a first-order Markovian process (switch models sensu Morales et al. [3]). Similar in logic to continuous-time movement models [4, 21, 22], we propose to look at patterns in the time spent within different modes, as well as transitions between modes, to refine classification of broad-scale movement strategies.

We focused on models of movement involving three modes, two corresponding to different areas of concentrated movement (equivalent to two encamped movement modes) and a third allowing for movement outside and between these areas (equivalent to an exploratory mode). We first investigated the model’s applicability using simulated data following four stereotypical movement strategies (migration, dispersal, nomadism, and sedentarism). We then applied our approach to a sample of GPS-tagged Galapagos tortoises (*Chelonoidis* sp.), with 8 individuals drawn from four species across three islands. Lastly, we introduced extensions of our approach that would allow for the addition of site-specific variables and enable assessment of classification robustness based on a bootstrap approach.

## Methods

### Model formulation and estimation

Net squared displacement is obtained from a series of locations by calculating the square of the Euclidean distance between a given location and the putative origin of a movement path. This distance is the straight line distance between the first location and each subsequent location along the time series [17]. We analyzed individual time series of NSD using a mixture-model including two normal and one uniform distribution. A normal distribution can be associated with areas of intensive and recurrent space-use (encamped mode), whereas the uniform distribution is related to sporadic use of an area while travelling (exploratory mode). Initially we were unsure if some movement types (e.g., home ranging or nomadic movement) might be well fit by a single normal and uniform mixture. Preliminary analysis suggested that a model with two normal distributions and a uniform distribution better fit simulated data from all movement strategies we considered. For a migrating animal, we expected the frequency distribution of NSD values (Fig. 1, first row) to be best described by a mixture of two normal distributions each associated with encamped movement within a seasonal range and having a uniform distribution representing the transition period between the two ranges. For a dispersing animal, we would expect a similar pattern in frequency (Fig. 1, second row), which would be distinguishable from migratory behavior if the animal does not transition back to the first encamped movement mode. Nomadic and sedentary behavior could be captured by a similar structure with multiple transitions between each mode (Fig. 1, third and fourth rows). In this case, we predict that sedentarism will involve numerous transitions between the two encamped modes while nomadic behavior will have fewer transitions.

A mixture-model can be formulated as a latent variable model where each observation, NSD_t_ (t = 1, …, T), is associated with an unobserved (latent) mode indicator variable I_t_ = i, i ∈ {1, …, M} where *M* is the number of different clusters and defined explicitly by the model structure. Starting with a simple case for a given vector of modes *I*, the likelihood function for a mixture of normal distributions for a set of observations *y* = {NSD_1_,…,NSD_T_} of NSD values is:

1 $$ L\left(y\left|\upmu, \sigma, I\right.\right)={\displaystyle {\prod}_{t=1}^TN\left(NS{D}_t\left|{\mu}_{It}\right.{\sigma}_{It}\right)} $$

where $N\left(NS{D}_t\Big|{\mu}_{I_t},{\sigma}_{I_t}\right)$ is the value at time *t* of the probability density function of a normal distribution with mean *μ*_*I*_ and standard deviation *σ*_*I*_. Since our framework requires the addition of a uniform distribution, we need to include this into eqn 1. It is computationally efficient and mathematically simple to approximate the model as a mixture of three normal distributions where one normal distribution replicates a uniform distribution (hereafter referred as pseudo-uniform). This is done by holding *μ* and *σ* fixed for *I = 3* in order to obtain a relatively flat distribution over the range of observed values for the third distribution. Hence, in eqn 1, ${\mu}_{I_t}$ and ${\sigma}_{I_t}$ are each estimated by the model for the first two modes, and fixed for the third mode.

Implicitly, eqn 1 assumes that the NSD at time *t* is independent of the NSD at time *t*-1, given the vector of modes, *I*. Temporal autocorrelation in NSD values is likely to be common for all movement strategies, but especially for dispersal and migratory strategies [23]. To better accommodate the temporal autocorrelation present in NSD time-series, we added parameters to describe the probability of switching from one mode to another in order to explicitly model the Markovian switch. At each time step, an individual changes from a current mode to another with fixed probability. For our model based on a mixture of two normal and pseudo-uniform distributions, we used a 3 × 3 matrix Q to define the probability, *q*_*ij,*_ of being in mode *i* at time *t* + 1 given that the individual is in mode *j* at time *t.* One advantage of this approach is that even when data are unavailable, the matrix of switching probabilities can assign a mode to every time step, therefore allowing straightforward modelling of missing data. The likelihood of this model is obtained by multiplying eqn 1 by the matrix Q (we present the full expression of the likelihood in Additional file 1). A Bayesian approach using Monte Carlo Markov-Chain (MCMC) techniques is the most common method used for estimating parameters when dealing with latent-state models. We developed the model in JAGS 3.4.0 [24] and created a series of functions for movement analysis in R (R Core Team 2015) available at https://github.com/BastilleRousseau/lsmnsd. To simplify the integration of priors, we range-standardized the NSD values across all values, such that values ranged from zero to one for each individual. Model structures are presented in Additional file 2. Unless otherwise specified, we conducted all analyses in R v 3.1.2 (R Core Team 2015).

### Simulated movement study

To test the proposed approach, we applied it to movement paths simulated from random walk data. We simulated movement associated with four strategies (nomadism, sedentarism, dispersal, and migration) for 365 time steps (i.e., 1 year of data with one location per day). Our simulated dataset is similar to that of Bunnefeld et al. [14], but we allowed movement parameters to vary to a greater degree to represent a wider range of movement trajectories. We used functions implemented in the R package adehabitatLT to simulate movement [25] and we provide a more in-depth description and associated scripts in Additional file 3. For each movement type, we generated 1000 simulated datasets, for a total of 4000 movement trajectories, each consisting of 365 time steps.

We applied the clustering algorithm on each of the simulated trajectories. We first tested for convergence using uninformative priors with three chains and 5000 iterations. For scenarios that did not converge, we reran with 20,000 iterations. Convergence was assumed when the Gelman-Rubin convergence statistic $\left(\hat{R}\right)$ < 1.1 for parameters related to deviance, mean and standard deviation of each mode, and switching probabilities. This statistic compares the within-chain variance of a parameter to the between-chain variance. We extracted the matrix of switching probabilities and also associated each location to its most probable mode based on the MCMC iterations. We then estimated the number of transitions between one mode and another. We used these summary statistics to determine criteria best suited to differentiating among each movement strategy. We used half of our simulated datasets to generate a set of simple rules that formed the basis of a classification system, and we determined the accuracy of these classification rules using the second half of the simulated datasets.

We also tested whether our approach outperformed other approaches by applying Bunnefeld et al.’s [14] method to our simulated movement trajectories. For each simulated time-series, we fitted a double sigmoid curve (migration), a sigmoid curve (dispersal), a linear model (nomadism), and a constant model (sedentarism) using non-linear modelling. We associated each trajectory to a strategy by looking at the top model based on AIC (see Bunnefeld et al. [14] for further details of the approach). Lastly, we calculated a kappa statistic [26] for each approach to assess overall agreement. We did not compare our approach with the approach suggested by Börger & Fryxell [15] since we were unable to obtain convergence of the double-sigmoid model in a mixed-effects framework because of the variability in our simulations (i.e. Börger & Fryxell [15] did not apply their approach to migration). We contend that this is a limitation of the Börger & Fryxell [15] approach – mixed-effects models may be hard to estimate in population with highly variable movement strategies.

### Application to giant tortoises

**Fig. 2:**
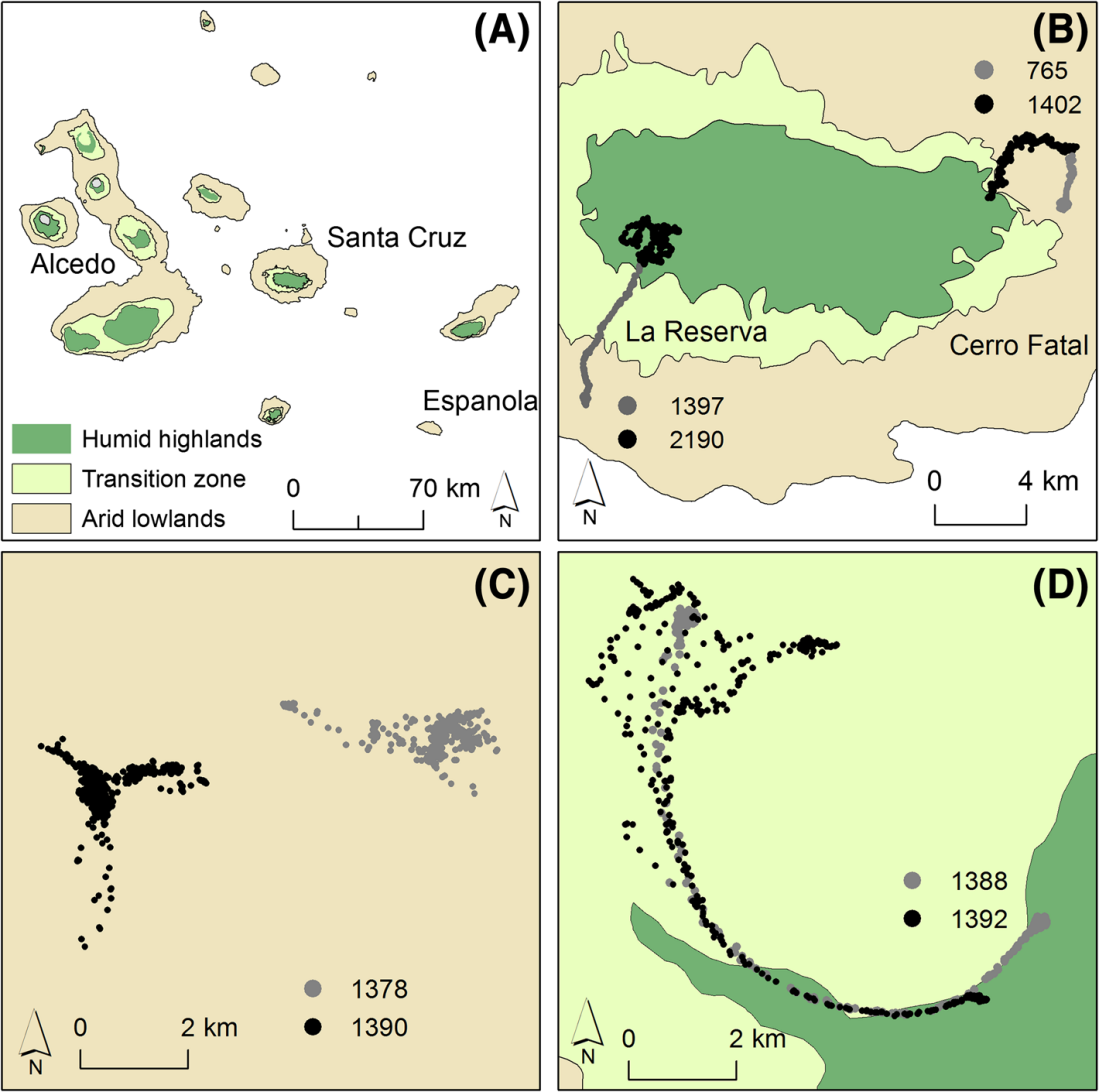
Population ranges of 83 giant tortoises of four different taxa inhabiting the Galapagos islands, 2009–2014. **a** The Galapagos Archipelago, illustrating vegetation zones, **b** Santa Cruz Island, including tortoise tracks and the Cerro Fatal and La Reserva regions, **c** Tortoise tracks on Espanola Island, **d** Tortoise tracks on Alcedo Volcano

Giant tortoises occur across six different islands throughout the Galapagos archipelago. We sampled tortoises movements in four species [27] across three islands including two tortoise morphotypes–“saddlebacks” (with elevated frontal portions of the carapace, which occur on arid low-lying islands) and “domes” (with carapaces that extend low over the head, which occur on islands with humid highlands; [26]). We attached custom-made GPS tags (e-obs, Munich, Germany) to 70 adult tortoises, and collected GPS locations from these tags between 2009 and 2014. This sample was comprised of 18 individuals (9 F, 9 M) on the relatively flat and arid Espanola Island, 11 individuals (6 F, 5 M) on Isabela Island, and 54 individuals on Santa Cruz Island, with 14 (7 F, 7 M) in the “*Cerro Fatal*” population and 40 (20 F, 20 M) in the “*La Reserva*” species (Fig. 2 [8]).

We attached custom-made GPS tags (e-obs, Munich, Germany) to 70 adult tortoises, and collected GPS locations from these tags between 2009 and 2014. Tortoises are largely immobile at night, so we programmed GPS units to record locations every hour during the day (5 AM – 7 PM) to maximize battery life. For our study, this resulted in a total of 911,018 locations (ranging from 78 to 26,788 locations per individual). We measured the size (curved carapace length) and sex of the individual when attaching tags. All animal handling procedures followed the guidelines of the Galapagos National Park, the Max Planck Institute of Ornithology, and IACUC protocol #121202 of the State University of New York, College of Environmental Science and Forestry.

### Analyzing tortoise movement

We applied our modelling approach to the multi-year movement data of eight giant tortoises. First, we simplified each individual trajectory by averaging the hourly locations for each day. This was necessary because we did not have continuous monitoring at night. This also improved analysis speed (analysis of a movement path with 25,000 iterations and 1500 locations was generally performed in less than 30 min on a 3.60 Ghz processor). Problems with some of the GPS tags led to the presence of gaps in the time series of NSD values. In a model based on a Markov process, the statistical state can still be assigned for days without NSD data (with associated uncertainty) based on the NSD values in surrounding days and the estimated transition matrix (see Additional file 3 for model structure). We ran the model using 3 chains and 25,000 iterations and confirmed convergence by $\hat{R}$ < 1.1. When convergence was not achieved (*n* = 10), we reran these models using 250,000 iterations and with different starting values. In a few cases (*n* = 3), it was also necessary to tighten the priors to facilitate convergence. Specifically, we tightened the range of the minimum and maximum values for the uniform distribution specified as the prior for *σ* which resulted in convergence for all scenarios. Lastly, we extracted the matrix of switching probabilities and the number of transitions between each mode. As the extent of monitoring was different among individuals, we scaled the total time spent in each mode and number of transitions between modes over the duration of a uniform period of 365 days.

### Model extensions

We present the details of two extensions to our approach. First, we examined whether using other statistics in addition to NSD improved our results (i.e., we extended our model in which latent state are inferred from a univariate statistics for a multivariate case). Second, we developed an approach (similar to a bootstrap) that tested how sensitive classification was to the starting point of a NSD time-series. We present the methodological details and results in Additional files 4 and 5, respectively.

## Results

### Simulated movement

Models fitted to simulated nomadic movement converged within 5000 iterations for 88 % of simulated datasets compared to >95 % of datasets for the other simulated strategies. Increasing the number of iterations to 20,000 resulted in >95 % convergence of analyses based on nomadic datasets and >99 % for other strategies.

**Fig. 3:**
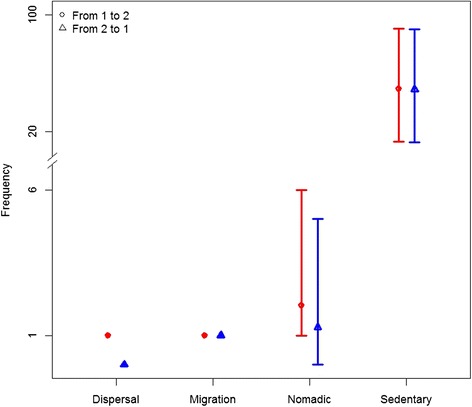
Number of transitions between encamped modes for simulated movement strategies. Legend: For each strategies and transitions, average and 95 % confidence intervals are presented. Note the broken y-axis. Strategies represented are dispersal, migration, nomadism, and sedentarism

We compared model characteristics that could potentially differentiate movement strategies. These included the matrix of switching probability and the number of transition between modes (Table 1 and Fig. 3). Simulated migration and dispersal movements had a very low frequency of switching between modes, as indicated from high probabilities along the matrix diagonal (Table 1); but were indistinguishable solely based on their matrix of switching probabilities. Sedentarism simulations were characterized by frequent transitions among modes as indicated from the low probabilities along the matrix diagonal (Table 1). Nomadism movement had intermediate probabilities of switching, particularly for the probability of staying within the uniform mode (*q*_*33*_) which was lower than for dispersal and migration (Table 1). The frequency of transitions between each mode was also a strong indicator of specific movement strategies. All simulated dispersal movement resulted in one observed transition from the first encamped mode to the second encamped mode and no transitions from the second to the first (Fig. 3). For simulated migratory movements, all observations followed the pattern of one transition from the first encamped mode to the second and one from the second encamped mode back to the first (Fig. 3). Nomadic movement simulations showed more frequent transitions in both directions; but generally < 10 (Fig. 3). Finally, sedentary simulations showed the number of transitions in both directions to be an order of magnitude higher than the other strategies (Fig. 3).

**Table 1:** Matrix of switching probabilities (q_ij_) based on latent-state modelling estimated from simulated movement (*n* = 500) associated to four strategies (dispersal, migration, nomadic, and sedentarism)

	j = 1	j = 2	j = 3
Dispersal			
i = 1	0.981 (0.965, 0.988)	0.006 (0.004, 0.012)	0.013 (0.008, 0.024)
2	0.006 (0.004, 0.012)	0.987 (0.976, 0.992)	0.006 (0.004, 0.012)
3	0.024 (0.015, 0.064)	0.044 (0.031, 0.079)	0.931 (0.866, 0.953)
Migration			
i = 1	0.98 (0.966, 0.987)	0.006 (0.004, 0.012)	0.013 (0.008, 0.023)
2	0.008 (0.005, 0.015)	0.977 (0.954, 0.985)	0.015 (0.01, 0.031)
3	0.026 (0.016, 0.057)	0.025 (0.016, 0.047)	0.948 (0.895, 0.968)
Nomadic			
i = 1	0.982 (0.952, 0.991)	0.012 (0.004, 0.041)	0.005 (0.004, 0.012)
2	0.014 (0.004, 0.049)	0.972 (0.927, 0.99)	0.012 (0.005, 0.028)
3	0.235 (0.028, 0.362)	0.323 (0.04, 0.396)	0.407 (0.304, 0.925)
Sedentarism			
i = 1	0.799 (0.502, 0.941)	0.191 (0.055, 0.477)	0.007 (0.003, 0.027)
2	0.522 (0.122, 0.831)	0.445 (0.141, 0.846)	0.03 (0.01, 0.068)
3	0.281 (0.077, 0.685)	0.423 (0.171, 0.684)	0.217 (0.094, 0.636)

Median probabilities with 95 % confidence intervals are provided. Switching probabilities are estimated based on a latent-state model including three movement modes, two associated to encamped movement (1 and 2) based on a normal distribution and a third exploratory mode (3) based on a pseudo-uniform distribution

### Potential rules for classification and comparison to alternative approaches

Using the matrix of switching probability as the basis for classification is overall preferable given that the metrics related to patterns in time spent and transitions between modes ignored location classification uncertainty. Yet, distinguishing between dispersal and migration also requires estimating if the individual stayed in or left the second mode. A series of simple rules are emerging from the variation observed in the matrix of switching probabilities (based on the estimated credible intervals, Table 1). Complementing these cut-offs with the presence of transition back from the second encamped mode allows correct classification of each movement strategies (Table 2). We classified movement as dispersal when *q*_*11*_ > 0.95, *q*_*22*_ > 0.95 and *q*_*33*_ > 0.85 and the simulated individual did not leave the second mode (M2, Table 2). We classified movement as migration when *q*_*11*_ > 0.95, *q*_*22*_ > 0.95 and *q*_*33*_ > 0.85 and the simulated individual left the second mode at least once. Movement was classified as nomadic when *q*_*11*_ > 0.95, *q*_*22*_ > 0.90 and *q*_*33*_ < =0.85 and sedentary when *q*_*22*_ < =0.90 and *q*_*33*_ < =0.90. Given the uncertainty inherent to classifying nomadic individual, we used conservative cut-off for this strategy, and characterized any simulation that did not fit the above criteria as “uncertain”, which would require secondary examination. Using only these criteria (Table 2) as the basis for classification outperformed the nonlinear modelling approach proposed by Bunnefeld et al. [14] for each strategy (kappa statistic = 0.93 vs 0.71).

**Table 2:** Proportion of simulated movement types classified into each movement based on simple set of rules

Predicted	Criteria		Dispersal	Migration	Nomadic	Home-ranging
	*P(q*_*ij*_ *)*	M_2_-M_1–3_				
Dispersal	*q*_*11*_ > 0.95 *q*_*22*_ > 0.95 *q*_*33*_ > 0.85	=0	0.98 (0.88)	0.00 (0.06)	0.06 (0.58)	0.00 (0.12)
Migration	*q*_*11*_ > 0.95 *q*_*22*_ > 0.95 *q*_*33*_ > 0.85	>0	0.00 (0.11)	0.99 (0.94)	0.07 (0.18)	0.00 (0.03)
Nomadic	*q*_*11*_ > 0.95 *q*_*22*_ > 0.90 *q*_*33*_ < =0.85		0.02 (0.01)	0.00 (0.00)	0.83 (0.20)	0.00 (0.00)
Home- ranging	*q*_*22*_ < =0.90 *q*_*33*_ < =0.90		0.00 (0.00)	0.00 (0.00)	0.01 (0.04)	1.00 (0.85)
Uncertain	Else		0.00	0.00	0.02	0.00

Criteria are based on the matrix of switching probability (representing the probability q of switching from mode i to j) and whether an individual departed the second encamped mode (M2). Numbers in parentheses reflect predicted strategies based on the non-linear modelling approach proposed by Bunnefeld et al. [14] applied to our simulated movement trajectories

### Application to giant tortoises

**Fig. 4:**
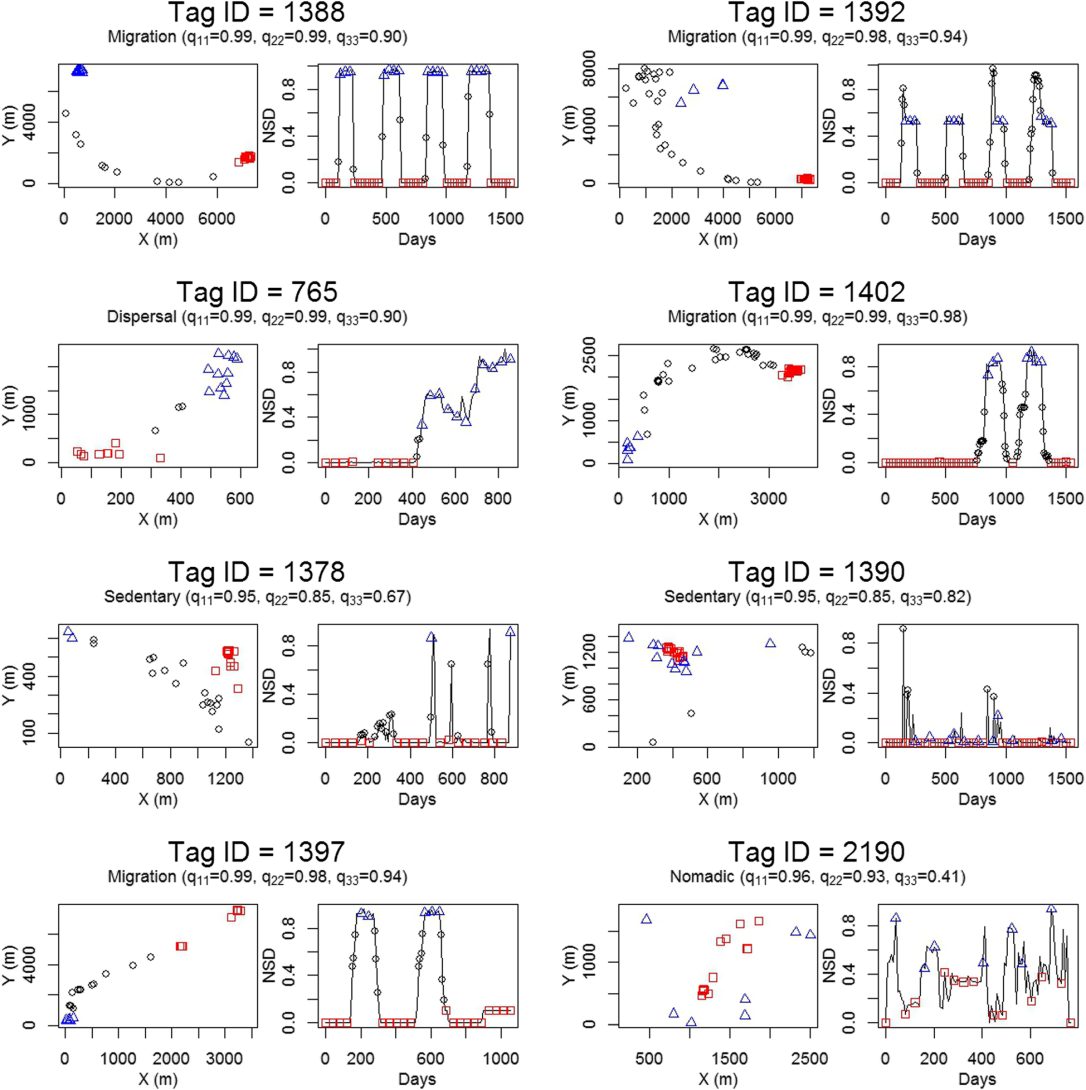
Examples of movement paths of 8 giant tortoises from four different species inhabiting the Galapagos islands, 2009–2014. Legend: For each tortoise, movement in the x-y plane and the corresponding pattern in NSD are presented. Relocations are color and shape associated with a specific mode. The first two individuals are from the Alcedo taxon, the second row is from the Cerro Fatal taxon, the third row are individuals in the Espanola taxon and the last row represents individuals from La Reserva. Switching probabilities *q*_*11*_, *q*_*22*_, and *q*_*33*_ are also presented to assist in classification of movement strategies

Our analysis revealed marked variation among individuals in their movement strategies (Fig. 4). For brevity, we focused the presentation of movement strategies on a subset of eight individuals (two from each species) that were representative of the larger dataset (Fig. 4). Four individuals analyzed showed patterns of transition between two movement modes indicative of migratory behavior (p_11_ > 0.95, p_22_ > 0.95, and p_33_ > 0.85). In these instances, individuals moved between two core areas, transitioning from one mode to the second and back an average of once per year (e.g., Tag ID 1388, 1392 and 1397 in Fig. 4). These individuals normally spent a substantial amount of time in the second mode. Among the migratory individuals, there was variability in the frequency and timing of migration among years, some individuals even remained in one range an entire year (e.g., Tag ID 1402 and 1397 in Fig. 4). One individual (e.g., Tag ID = 765 in Fig. 4) did not transition back to its original mode, this was indicative of dispersal behavior.

We found two individuals with patterns of transition indicative of a sedentarism strategy (e.g., Tag ID 1378 and 1390 in Fig. 4). This strategy was characterized by values of *q*_*22*_ < 0.90 and *q*_*33*_ < 0.90. Interestingly, Tag 1378, had an annual average number of transitions similar to a migratory pattern, but spent a very short time in the second mode relative to the first one. In such instances, a pattern label akin to an “exploratory resident” strategy might be more appropriate. Finally, one individual displayed nomadic behavior (*q*_*11*_ > 0.95, *q*_*22*_ > 0.90 and *q*_*33*_ < =0.85, Tag ID 2190 in Fig. 4).

A multivariate version of the model took more iterations to converge and yielded similar results to the univariate version based solely on NSD in the classification of individual (Additional file 4), however this approach may be useful in certain applications. The classification of movement strategies in giant tortoises was weakly influenced by the starting date of the time-series of NSD; but overall; most classifications were insensitive to a change in starting date (Additional file 5).

## Discussion

Both our simulations and empirical analysis showed that switching probabilities estimated from a latent-state model can form the basis of a simple system for the assignment of movement strategies to individual animal trajectories. Our classification approach performed extremely well for simulated migration, dispersal, and sedentary movement, and greatly improved over past methods with respect to nomadic movement. The accuracy of our method in classifying each strategy was higher than classification accuracy reported by Bunnefeld et al. [14] and Börger & Fryxell [13] for their NSD approaches and our approach also directly outperformed the Bunnefeld et al. [14] approach when applied to our simulated movement. Our increase in classification accuracy is particularly noteworthy given that we used a wider range of parameters to initiate our simulations, and therefore generated paths with greater overlap in movement parameters compared to previous studies.

Instead of fitting idealized curves to how we expect animals to move between discrete regions of space (i.e., seasonal ranges), our approach expected those regions to manifest in concentrated movements punctuated by more or less frequent transitions for different strategies. Our approach greatly improved the classification of movement strategies for giant tortoises compared to previous analyses based on curve-fitting approaches. A past study focused on two species of tortoises found on Santa Cruz Island [8]. Here, we analyzed longer time-series and additional individuals from these species and added movement data from two other species found on Espanola Island and the Alcedo volcano on Isabela Island respectively (Fig. 2). Consistent with previous findings, we observed marked inter-individual variation in movement strategies of giant tortoises and found strong evidence of annual migrations on Santa Cruz Island [8]. Many individuals on the Alcedo volcano on Isabela Island also underwent an annual migration, while we found no evidence of migration on Espanola Island – the only island lacking substantial spatial variation in environmental conditions and vegetative communities.

Many tortoises, including the majority of tortoises on Espanola, were categorized as sedentary. In these cases, tortoises had an area associated with one movement mode with short expeditions (<15 days) outside of this area. Our analysis revealed variation in the frequency and duration of these “extra-home-range” expeditions. Some individuals made a few short trips outside their core ranges, a strategy we termed exploratory resident. Others made numerous shorter trips (>10 per year), a strategy more likely exhibited by individuals using a central-place foraging strategy [28]. By providing information related to residency time and frequency of transitions among different areas, our approach revealed complex patterns of movement strategies, even within the group of tortoises normally considered as non-migratory.

## Methodological improvement over previous alternatives

Our approach applied an established statistical framework, discrete latent-state modelling, to improve classification of movement strategies using NSD. We used NSD, instead of turning angles or step length [3], as the metrics at the basis of the movement classification. While step length and turning angles are accurate descriptors of fine-scale movement patterns, NSD has been shown as the statistic that best captures broad-scale patterns [16]. In a migration context, using NSD offers an objective way to spatially and temporally delineate modes corresponding to core range use as well as the mode associated to the migratory journey.

Our approach offers a number of advantages over alternative approaches previously used. Firstly, our approach account for temporal autocorrelation in NSD time-series, whereas past approaches did not account for this important feature of the data. Adding a matrix of switching probability to our approach addressed this concern. Secondly, while the use of the concordance criterion as suggested by Börger & Fryxell [13] may be best suited to assess fit for non-linear models [29], this criterion does not include a penalty term for the number of parameters and, as a result, increases the likelihood that more complex models (e.g., models indicating migration and dispersal) will be retained. In addition, the concordance criterion is similar to the R^2^, in that it converges toward zero (indicating lower fit) when assessing an intercept only model (which is often used to represent sedentarism behavior; [6, 14]). These two elements could have driven Singh et al. [7] to only classify 2 % of moose as residents while previous studies have found this strategy to be more common [30].

Our approach is also flexible regarding the data inputs and movement strategies it can characterize. For example, we made fewer assumptions about the seasonality of migration and we did not restrict migratory movements to an annual or even a regular basis. This made our approach extremely well suited for long-term datasets where individuals are monitored for multiple years, accommodating typical variation in the extent of individual monitoring, and revealing partial migratory tendencies. This flexibility does come at a cost in that our approach is not as automatic as that suggested by Bunnefeld et al. [14]. Our rules for classification are also best suited for daily locations, not resampling movement trajectories at the daily scale may requires small adjustment to the cut-offs used. In order to simplify the estimation process by having to manually set the priors, we also used range-standardized NSD time-series, which limits the comparison and interpretation of the outputs produced by the functions, especially among individuals. It is, however, possible to obtain other information by associating actual GPS locations with their corresponding mode *a posteriori*, making it possible to quantify distance travelled and timing of migration at the scale of the individual locations [15]. It is also possible to apply the inverse of the range-standardization to posterior distribution of NSD values in each mode. Assigning GPS relocations to modes also might improve subsequent analyses investigating potential differences in movement attributes or resource selection patterns [3]. Nonetheless, for many species and research questions, the large quantity of information our analysis provides outweighs the simplicity of previous approaches, and also avoids becoming a “black box” approach.

## Conclusions

Advances in our ability to track animal movement coupled with greater availability and resolution of environmental data offers new opportunities for movement ecologists [31, 32]. However, the spatial and temporal complexity of environmental data and the variability of animal movements make defining movement strategies and identifying their causes and consequences complicated. Following the movement ecology paradigm proposed by Nathan et al. [1], a fundamental first step in studying animal movement is to identify different movement strategies (e.g., distinguishing between stable range use and migratory or dispersing events; [33]). However, the movement patterns of individual animals are often difficult to objectively classify into particular strategies. Here, we expanded on previous approaches that used NSD to distinguish between several movement strategies including migration, dispersal, nomadism and sedentarism [7, 13, 14] by using a latent-state modelling. Whereas visual assessments of the NSD time series may be subjectively used to classify movement strategies, our approach based on mixture-modelling provides a rigorous framework that can be applied across different sampling schemes, and by extension can offer greater comparability of classified movement strategies.

## Availability of supporting data

The data supporting the results of this article are available and freely available on Movebank (Galapagos Tortoise Movement Ecology Programme; www.movebank.org). Functions for R are also available on GitHub at https://github.com/BastilleRousseau/lsmnsd.

## Additional files

Additional file 1: Full derivation of the likelihood. (DOCX 20 kb) Additional file 2: Scripts for clustering. (DOCX 20 kb) Additional file 3: Description of simulated movement strategies. (DOCX 22 kb) Additional file 4: Multivariate clustering. (DOCX 149 kb) Additional file 5: Influence of starting date. (DOCX 446 kb)
