# Supplementary material for: Flexible characterization of animal movement pattern using net squared displacement and a latent state model
Source: Mov Ecol. 2016 Jun 1;4:15. doi: 10.1186/s40462-016-0080-y (PMC4888472; doi:10.1186/s40462-016-0080-y)
Supplement: Additional file 1: — Full derivation of the likelihood. (DOCX 20 kb) [file 40462_2016_80_MOESM1_ESM.docx]

**Additional file 1: Full derivation of the likelihood**

We added parameters to describe the probability of switching from one normal distribution to another, to explicitly model the switch from one mode to another. At each time step, an individual changes from a current mode to another with fixed probability. For a bi-modal model based on a mixture of two normal and a pseudo-uniform distributions, we used a 3 x 3 matrix Q=(q_ij_) to define the probability, *q_ij,_* of being in mode *i* at time *t* + 1 given that the individual is in mode *j* at time *t.* The likelihood expression is then:

$L\left( y | \mu, \sigma,Q \right)=\left( \begin{matrix} p_{1} & p_{2} & 1-p_{1}-p_{2} \end{matrix} \right)M\left( {NSD}_{1} \right)\prod_{t=2}^{T} \left[ QM\left( {NSD}_{t} \right) \right]\left( \begin{matrix} 1 \\ 1 \\ 1 \end{matrix} \right),$ eqn 1

where $M\left( {NSD}_{t} \right)$ is the diagonal matrix whose entries correspond to the three cluster distributions

$M\left( {NSD}_{t} \right)=\left( \begin{matrix} N\left( {NSD}_{t} | \mu_{1},\sigma_{1} \right) & 0 & 0 \\ 0 & N\left( {NSD}_{t} | \mu_{2},\sigma_{2} \right) & 0 \\ 0 & 0 & N\left( {NSD}_{t} | \mu_{3},\sigma_{3} \right) \end{matrix} \right),$ eqn 2

and $p_{1}$ is the probability that the animal started in mode 1 and p2 that the animal started in mode 2.
