# Supplementary material for: Flexible characterization of animal movement pattern using net squared displacement and a latent state model
Source: Mov Ecol. 2016 Jun 1;4:15. doi: 10.1186/s40462-016-0080-y (PMC4888472; doi:10.1186/s40462-016-0080-y)
Supplement: Additional file 2: — Scripts for clustering. (DOCX 20 kb) [file 40462_2016_80_MOESM2_ESM.docx]

**Additional file 2: Scripts for clustering**

M1 <- 'model {

#Priors for the mean of each cluster

mu[1] ~ dunif(mu1.min,mu1.max) #

mu[2] <- min(mu[1] + eps[1], 1)

mu[3] <- 0.5 # Uniform distribution

eps[1]~dunif(mu2.min,mu2.max)

# Priors for the standard deviation

sigma[1] ~ dunif(sigma1.min,sigma1.max)

sigma[2] ~ dunif(sigma2.min,sigma2.max)

sigma[3]<-10 # Large standard deviation to create uniform distribution

# Precisions

tau[1] <- pow(sigma[1],-2)

tau[2] <- pow(sigma[2],-2)

tau[3] <- pow(sigma[3],-2)

#Probability for first location

alpha1[1] <- 1

alpha1[2] <- 1

alpha1[3] <- 1

p.state[1:3] ~ ddirch(alpha1[])

#Probability matrix

idx[1] ~ dcat(p.state[])

alpha[1]<-1

alpha[2]<-1

alpha[3]<-1

trans.mat[1,1:3]~ddirch(alpha[])

trans.mat[2,1:3]~ddirch(alpha[])

trans.mat[3,1:3]~ddirch(alpha[])

pi<-3.141593

# State assignation for each time-step

for (t in 2:ndays){

idx[t]~dcat(trans.mat[idx[t-1],])

}

#

for (i in 1:npts) {

# Loglik of each iteration - necessary for the WAIC

loglik[i] <- -0.5*log(2*pi*sigma[idx[day[i]]]) - (NSD[i]-mu[idx[day[i]]])/(2*sigma[idx[day[i]]])

# Mixture model

NSD[i]~dnorm(mu[idx[day[i]]],tau[idx[day[i]]])

}

}'
