# Supplementary material for: Flexible characterization of animal movement pattern using net squared displacement and a latent state model
Source: Mov Ecol. 2016 Jun 1;4:15. doi: 10.1186/s40462-016-0080-y (PMC4888472; doi:10.1186/s40462-016-0080-y)
Supplement: Additional file 3: — Description of simulated movement strategies. (DOCX 22 kb) [file 40462_2016_80_MOESM3_ESM.docx]

**Additional file 3: Description of simulated movement strategies**

**Migration (Figure 1, first row)**

1. We simulated the initial seasonal-range using a 2-D Ornstein-Uhlenbeck process with symmetric attraction (ranging from 0.05 to 1) and noise matrices (ranging from 10 to 1000).
2. We added a Brownian bridge model to represent the first migratory period and we randomly selected the migratory period duration (30 to 70 time steps).
3. The second seasonal-range was again simulated using an Ornstein-Uhlenbeck with the attraction centred on the ending location of the migratory period. We randomly selected the period spent in the second home-range (between 50 to 200 days) which was centred on step 182.
4. We added a second migratory process with an additional Brownian bridge model (of 30-70 steps) oriented toward the origin where we simulated a third Ornstein-Uhlenbeck process.

**Dispersal movement (Figure 1, second row)**

1. We simulated the initial home-range using a 2-D Ornstein-Uhlenbeck process with symmetric attraction (ranging from 0.05 to 1) and noise matrices (ranging from 10 to 1000).
2. We added a Brownian bridge model to represent the dispersal period. We randomly selected the dispersal period duration (30 to 70 time steps), the middle point of the dispersal period (between steps 100 to 265), the distance travelled based on the duration of the dispersal period, a step length distribution factor (7.5 to 750), and a random angle to define the initial and ending locations used by the Brownian bridge model.
3. The settlement phase was again simulated using an Ornstein-Uhlenbeck with the attraction centred on the ending location of the dispersal period.

**Nomadic movement type (Figure 1, third row)**

1. We simulated movement using a random walk scaled around a median step length (scaling factor ranged randomly from 10 to 1000) for a given simulation.

**Home-range movement (Figure 1, fourth row)**

1. We used a 2-D Ornstein-Uhlenbeck process with symmetric attraction (ranging from 0.05 to 1) and noise matrices (ranging from 10 to 1000) to simulate home-range movement.

**R scripts (required the package adehabitatLT):**

## Resident - Home-range

a<-sample(seq(0.05,1, by =0.001), 1)

h<-s<-sample(10:1000, 1)

resid <- (simm.mou(date=1:365, a=diag(a,2), sigma=diag(s,2)))

## Nomadic - Brownian motion

noma<-(simm.brown(1:365, h=h))

#Dispersal

date1<-sample(100:265, 1)

duration1<-sample(30:70, 1)

dist_disp1<-duration1*h *0.75

hr <- simm.mou(date=1:(date1-duration1/2), a=diag(a,2), sigma=diag(s,2))

begin<-c(tail(ld(hr)$x,1), tail(ld(hr)$y,1))

angle<-sample(-pi:pi, 1)

end<-c(begin[1]+dist_disp1*cos(angle),begin[2]+dist_disp1*sin(angle))

bb<-simm.bb(date = (date1-duration1/2):(date1+duration1/2), begin = begin, end = end)

hr2 <- simm.mou(date=(date1+duration1/2):366, b=end, a=diag(a,2), x0=end, sigma=diag(s/2.5,2))

disp<-rbind(ld(hr), ld(bb)[-1,],ld(hr2)[-1,])

disp2<-as.ltraj(disp[,1:2], disp[,3], id="id")

#Migration

duration_plateau<-sample(50:200, 1)

begin_plateau<-182-(duration_plateau/2)

end_plateau<-182+(duration_plateau/2)

duration<-sample(30:70, 1)

dist_disp<-(duration*h)*0.75

hr <- simm.mou(date=1:(begin_plateau-duration), a=diag(a,2), sigma=diag(s,2))

begin<-c(tail(ld(hr)$x,1), tail(ld(hr)$y,1))

angle<-sample(-pi:pi, 1)

end<-c(begin[1]+dist_disp*cos(angle),begin[2]+dist_disp*sin(angle))

bb<-simm.bb(date = (begin_plateau-duration):begin_plateau, begin = begin, end = end)

hr2 <- simm.mou(date=begin_plateau:end_plateau, b=end, a=diag(a,2), x0=end, sigma=diag(s/2,2))

bb2<-simm.bb(date = end_plateau:(end_plateau+duration), begin = as.numeric(tail(hr2[[1]][1:2],1)), end = c(0,0))

hr3<-simm.mou(date=(end_plateau+duration):366, a=diag(a,2), sigma=diag(s,2))

mig<-rbind(ld(hr), ld(bb)[-1,],ld(hr2)[-1,],ld(bb2)[-1,],ld(hr3)[-1,])

mig2<-as.ltraj(mig[,1:2], mig[,3], id="id")
