# Supplementary material for: Flexible characterization of animal movement pattern using net squared displacement and a latent state model
Source: Mov Ecol. 2016 Jun 1;4:15. doi: 10.1186/s40462-016-0080-y (PMC4888472; doi:10.1186/s40462-016-0080-y)
Supplement: Additional file 4: — Multivariate clustering. (DOCX 149 kb) [file 40462_2016_80_MOESM4_ESM.docx]

**Additional file 4: Multivariate clustering**

We expanded our latent-variable model by adding a second normal distribution associated with a potential new variable and expanding the vector of NSD values *y* to a two columns matrix. For a given vector of modes *I* and given data *y*, the likelihood function analogous to equation 1 of the manuscript is expanded to:

$L\left( y | \mu_{1},\sigma_{1},\mu_{2},\sigma_{2} \right)=\prod_{t=1}^{T} N_{1}(n_{1_{t}}|\mu_{{1I}_{t}},\sigma_{{1I}_{t}})N_{2}(n_{2_{t}}|\mu_{{2I}_{t}},\sigma_{{2I}_{t}})$ eqn 5

Where $N_{1}(n_{1_{t}}|\mu_{{1I}_{t}},\sigma_{{1I}_{t}}) N_{2}(n_{2_{t}}|\mu_{{2I}_{t}},\sigma_{{2I}_{t}})$ is the value at time *t* of the probability density function of the two independents normal distribution with mean *µ1_I_* and standard deviation *σ1_I_* for the first variable (*n_1_*=NSD) and with mean *µ2_I_* and standard deviation *σ2_I_* for the second variable (*n_2_*= elevation or daily distance).

We tested this new formulation separately with two different variables – daily distance travelled and elevation. We expected an increase in distance traveled when individuals were migrating or dispersing. Distance was calculated from rediscretized movement trajectories. Moreover, previous studies have shown that altitudinal gradients may impact the presence of migratory movements of Galapagos tortoises in some populations (Blake *et al.* 2012). We ran the model using 3 chains and 25,000 iterations and assessed convergence by using $\hat{R}$< 1.1. All analyses were run using R v3.1.2.

With 25,000 iterations, adding a second variable to the clustering algorithm considerably reduced convergence. Using daily distance traveled as a second variable reduced convergence to 74% of the individuals analyzed, whereas including elevation further reduced convergence to 61% of the individuals. For models that converged, the resulting classification was highly similar to the univariate classification but sometimes worse (Figure S1). We failed to detect instances where multivariate clustering significantly improved our understanding and characterization of movement strategies and therefore we did not pursue assessing convergence for all individuals.


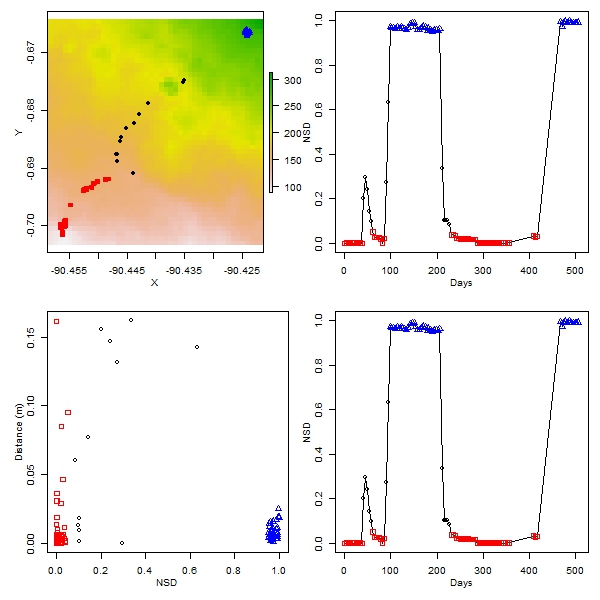


*Figure S1. Movement pattern of a giant tortoise (Tag ID = 1406) from La Reserva taxon. The upper row represented movement pattern in response to elevation in the x-y plan and the corresponding pattern in NSD over time. Relocations are colour-associated with a specific cluster based on bivariate clustering integrating NSD and elevation. The lower row represents the relationship between the NSD and daily distance travel with associated temporal pattern of NSD. Relocations are colour-associated with a specific cluster based on bivariate clustering integrating NSD and daily distance travel. Reader can refer to Figure 3 for a comparison with the univariate approach for this individual.*
