# Supplementary material for: Flexible characterization of animal movement pattern using net squared displacement and a latent state model
Source: Mov Ecol. 2016 Jun 1;4:15. doi: 10.1186/s40462-016-0080-y (PMC4888472; doi:10.1186/s40462-016-0080-y)
Supplement: Additional file 5: — Influence of starting date. (DOCX 446 kb) [file 40462_2016_80_MOESM5_ESM.docx]

**Additional file 5: Influence of starting date**

We developed an approach that tested how sensitive classification was to the starting point of a NSD time-series. We created a function that takes several windows of locations of shorter period. The width of the window and number of iterations (number of windows sampled) is defined by the interval in the starting date tested and the lag between the start of each window. These two parameters are specified by the user. The function then applies the clustering algorithm independently to each set of location and associates the predicted cluster to each location. It is then possible to extract percentage of agreement for a given location for the different iterations and also calculate an average agreement for a given dataset.

We tested the behaviour of this function for each tortoise by varying the starting date over the first 100 days of locations and by step of 7 days, leading to 15 iterations for every individual. The width of the window is maximised based on the number of locations available for an individual. In most situations, we expect that varying the starting locations by a shorter period would be more appropriate. We only ran models using 3 chains and 25,000 iterations and assessed convergence by using $\hat{R}$< 1.1.

Only 10 individuals (out of 70) had a percentage of iterations converging lower than 66% while 40 individuals had convergence rate higher than 93%. The different iterations revealed variation in the sensitivity of the classification to the starting date. On average, overall agreement for a classification was around 88% (median = 93%) but certain individuals had overall agreement < 62%. At the daily level, 72% of monitoring days had > 80% agreement (range: 12 - 100) among the different iterations. This value decreased to 57% (range: 6 – 100) when looking at the percentage of days with > 95% agreement among iterations. Overall, NSD patterns that can be associated to home-ranging (frequent transitions between the two clusters) had a lower agreement than patterns associated to migratory strategy, except when they stayed less than 100 consecutive days in the initial cluster (Figure S2). We recommend using the bootstrap approach to detect classifications that may be sensitive to the starting locations and requires further investigation, but suggest that a low percentage of agreement may not indicate problems with the actual classification.

**
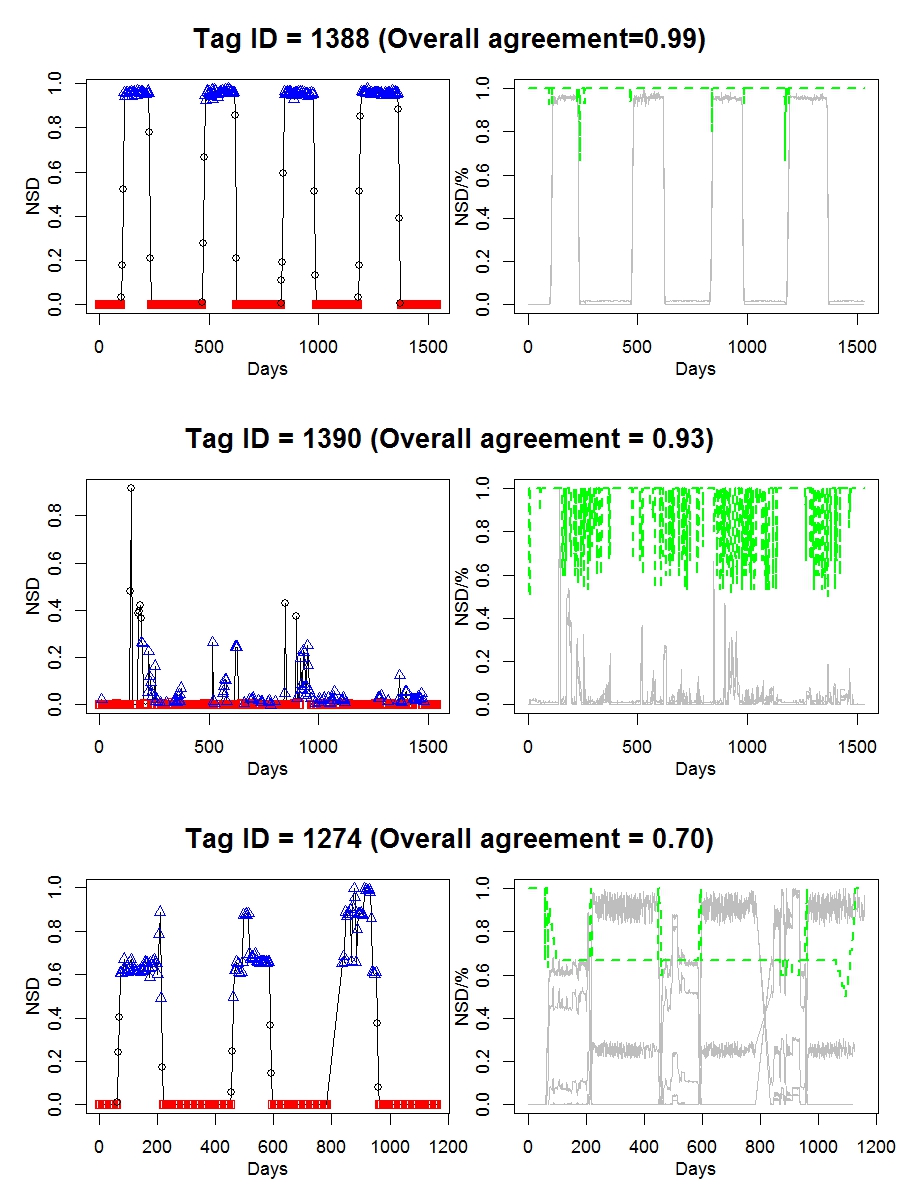
**

*Figure S2. Movement patterns of three giant tortoises. The left column represents the corresponding pattern in NSD over time with relocations colour-associated with a specific cluster based on univariate clustering. The right column represents NSD patterns over with different starting dates (n=15). The green line presents the percent of agreement amount the different iterations for each date.*
